# Pain catastrophizing, neuroticism, fear of pain, and anxiety: Defining the genetic and environmental factors in a sample of female twins

**DOI:** 10.1371/journal.pone.0194562

**Published:** 2018-03-22

**Authors:** Andrea Burri, Soshiro Ogata, David Rice, Frances Williams

**Affiliations:** 1 Health and Rehabilitation Research Institute, Auckland University of Technology, Auckland, New Zealand; 2 European Institute for Sexual Health, Hamburg, Germany; 3 Channing Division of Network Medicine, Department of Medicine, Brigham and Women’s Hospital and Harvard Medical School, Boston, United States of America; 4 Department of Health Promotion Science, Osaka University Graduate School of Medicine, 1–7 Yamadaoka, Suita, Osaka, Japan; 5 Research Fellow of Japan Society for the Promotion of Science, Tokyo, Japan; 6 Department of Twin Research and Genetic Epidemiology, King’s College London, London, United Kingdom; Brown University, UNITED STATES

## Abstract

The objective of the present study was to establish the heritability of pain catastrophizing and its subdomains of helplessness, magnification, and rumination and to further explore the genetic and environmental sources that may contribute to pain catastrophizing as well as to its commonly reported psycho-affective correlates, including neuroticism, anxiety sensitivity, and fear of pain. N = 2,401 female twin individuals from the TwinsUK registry were subject to univariate and multivariate twin analyses. Well validated questionnaires including the Pain Catastrophizing Scale, the Pain Anxiety Symptom Scale, the Ten Item Personality Index, and the Anxiety Sensitivity Index were used to assess the study variables. Moderate estimates of heritability for pain catastrophizing (36%) and the three subdomains of helplessness (35%), rumination (27%), and magnification (36%) were detected. The high correlations observed between the three subdomains were explained mainly by overlapping genetic factors, with a single factor loading on all three phenotypes. High genetic correlations between pain catastrophizing and its psycho-affective correlates of fear of pain and anxiety sensitivity were found, while the genetic overlap between neuroticism and pain catastrophizing was low. Each measure of negative affect demonstrated relatively distinct environmental contributing factors, with very little overlap. This is the first study to show shared genetic factors in the observed association between pain catastrophizing and other measures of negative affect. Our findings provide deeper insight into the aetiology of pain catastrophizing and confirm that it is at least partially distinct from other measures of negative affect and personality that may influence the development and treatment of chronic pain conditions. Further research in males is warranted to check the comparability of the findings.

## Introduction

Pain catastrophizing comprises a set of negative emotional and cognitive responses to pain and is thought to be made up of three dimensions: helplessness, magnification, and rumination, all of which are conceptually related and therefore show high correlations with one another [1]. Pain catastrophizing has emerged as one of the most robust psychological predictors of adverse pain outcomes and has been repeatedly associated with increased sensitivity to pain, increased risk of persistent pain, heightened pain intensity and severity, increased disability, and higher levels of psychological distress and depressive symptoms [1–7]. A systematic literature review by Sullivan and colleagues further reported that pain catastrophizing accounted for up to 31% of the variance in pain severity and, more importantly, that the correlation with disability was independent of the contribution of pain severity [1].

Several psychological factors have been shown to be linked to catastrophizing such as personality (i.e., neuroticism), negative affect, and certain sickness-related beliefs (i.e., about the organic origins of pain) [8–12]. In this regard, personality is not only related but can moderate the negative influence of catastrophizing on pain related outcomes, as has been shown to be the case with neuroticism [9]. Furthermore, the construct of negative affect is regarded as part of a higher order vulnerability factor influencing, for example, anxiety sensitivity and fear of pain, which might fuel catastrophizing tendencies [13]. Theories using a cognitive-behavioural framework have also suggested an important role of operant learning and social learning models in the development of catastrophizing. According to these models, individuals may have a heightened pain experience that then leads to increasingly pessimistic beliefs and decreased faith in their ability to cope with pain which in turn may aggravate the pain experience [1,7,14,15]. Conversely, other studies have provided evidence that catastrophizing tendencies can be manifested relatively early in life and predict pain outcomes even in the absence of major prior pain experiences, suggesting a potential involvement of genetic factors [16,17] Indeed, a number of recent studies have provided indirect evidence for a familial contribution to pain catastrophizing [18–20]. In the only twin study reported so far, Trost and colleagues examined pain catastrophizing in a sample of US twins and found a heritability of 37%, with the remaining 63% of the variance being explained by unique environmental influences [21]. In their study, however, the authors did not independently assess the heritability of the three sub-dimensions of pain catastrophizing (i.e., helplessness, magnification of pain, and rumination). A number of studies have confirmed such a three factor structure for pain catastrophizing, showing it to be invariant across current pain status [22], age [23], gender [24], and culture [25]. There is growing evidence that the three different subdomains of pain catastrophizing have differential influence on pain and disability [26] and preliminary evidence of different genetic contributions to each subdomain [27]. Thus, further exploration of the heritability and aetiology of pain catastrophizing and its subdomains is warranted.

Furthermore, the study by Trost and colleagues failed to explore the genetic or environmental overlap between pain catastrophizing and phenotypically related constructs such as neuroticism or other pain related measures of negative affect. This may be important, as a number of studies have demonstrated significant shared variance between catastrophizing and other measures such as fear of pain [28], anxiety [29] and neuroticism [9], suggesting possible construct redundancy and questioning the extent to which pain catastrophizing is unique or conceptually distinct from other measures of negative affect.

Thus, the aims of this study were to first establish the heritability of pain catastrophizing and its subdomains of helplessness, magnification, and rumination in a sample from TwinsUK. Second, we explored whether and to what extent the strong phenotypic correlation between the three subdomains of pain catastrophizing were due to common genetic and environmental factors. Third, we aimed to explore shared etiologic mechanisms of several other measures of negative affect and personality previously shown to be associated with pain catastrophizing, namely neuroticism, anxiety sensitivity and fear of pain.

## Results

Table 1 provides a summary of the study variables for the overall female sample and by zygosity. Significant differences between MZ and DZ twins could be found for age, with DZ twins being significantly older compared to MZ twins (62.4 vs. 56.2 years, p<0.001). Twins also differed significantly in terms of levels of magnification which were higher in MZ compared to DZ twins (2.1 vs. 1.9, p<0.05). Pain catastrophizing correlated significantly with all the study variables including anxiety sensitivity (r = 0.37), fear of pain (r = 0.38), and neuroticism (r = 0.19; p < .001 for all). Furthermore, the PCS subdomains of helplessness, magnification and rumination showed very high correlations with one another, ranging from r = .73 for rumination and magnification to r = .78 for helplessness and rumination (Table 2).

**Table 1:** Sociodemographic characteristics and main study variables of the overall sample (N = 2,401) and by zygosity.

	Full sample (N = 2,401)		MZ (N = 1,491)		DZ (N = 910)			
	N	%	N	%	N	%		
	M	SD	M	SD	M	SD	z	*P*
Age	58.49	14.01	56.23	14.92	62.19	11.45	-9.441	0.000**
PCS total	9.48	9.11	9.74	9.37	9.04	8.66	1.325	0.186
Rumination	3.72	3.57	3.80	3.67	3.59	3.41	0.923	0.356
Magnification	1.95	2.09	2.03	2.15	1.80	1.96	2.246	0.025*
Helplessness	3.80	4.26	3.90	4.36	3.64	4.06	0.892	0.373
Anxiety Sensitivity	13.38	8.85	13.08	8.50	13.77	9.27	-1.123	0.261
Neuroticism	3.25	1.41	3.26	1.40	3.24	1.41	0.412	0.680
Fear of pain	4.02	4.09	3.99	4.13	4.06	4.03	-0.676	0.499

* p-value < .05

** p-value < 0.001

M = Mean; SD = Standard Deviation; PCS = Pain Catastrophizing Scale.

**Table 2:** Phenotypic correlations between the main study variables (N = 2,401).

	PCS total	Rumination	Magnification	Helplessness	Anxiety Sensitivity	Neuroticism	Fear of pain
PCS total	-	-	-	-	-	-	-
Rumination	0.92*	-	-	-	-	-	-
Magnification	0.86*	0.73*	-	-	-	-	-
Helplessness	0.94*	0.78*	0.74*	-	-	-	-
Anxiety Sensitivity	0.37*	0.32*	0.36*	0.32*	-	-	-
Neuroticism	0.19*	0.17*	0.18*	0.19*	0.35*	-	-
Fear of pain	0.38*	0.32*	0.42*	0.35*	0.58*	0.26*	-

* p-value < .001; PCS = Pain Catastrophizing Scale.

### Univariate heritabilities

Univariate heritability (95% CI) for the total pain catastrophizing score was 36% (95% CI 29% to 42%). Heritability estimates for the subdomains were helplessness = 35% (95% CI 28% to 4%) rumination = 27% (95% CI 19% to 34%) and magnification = 36% (95% CI 28% to 42%). No influence of C or D could be detected for total pain catastrophizing or any of its subdomains.

### Multivariate modelling of the three pain catastrophizing subscales

**Fig 1:**
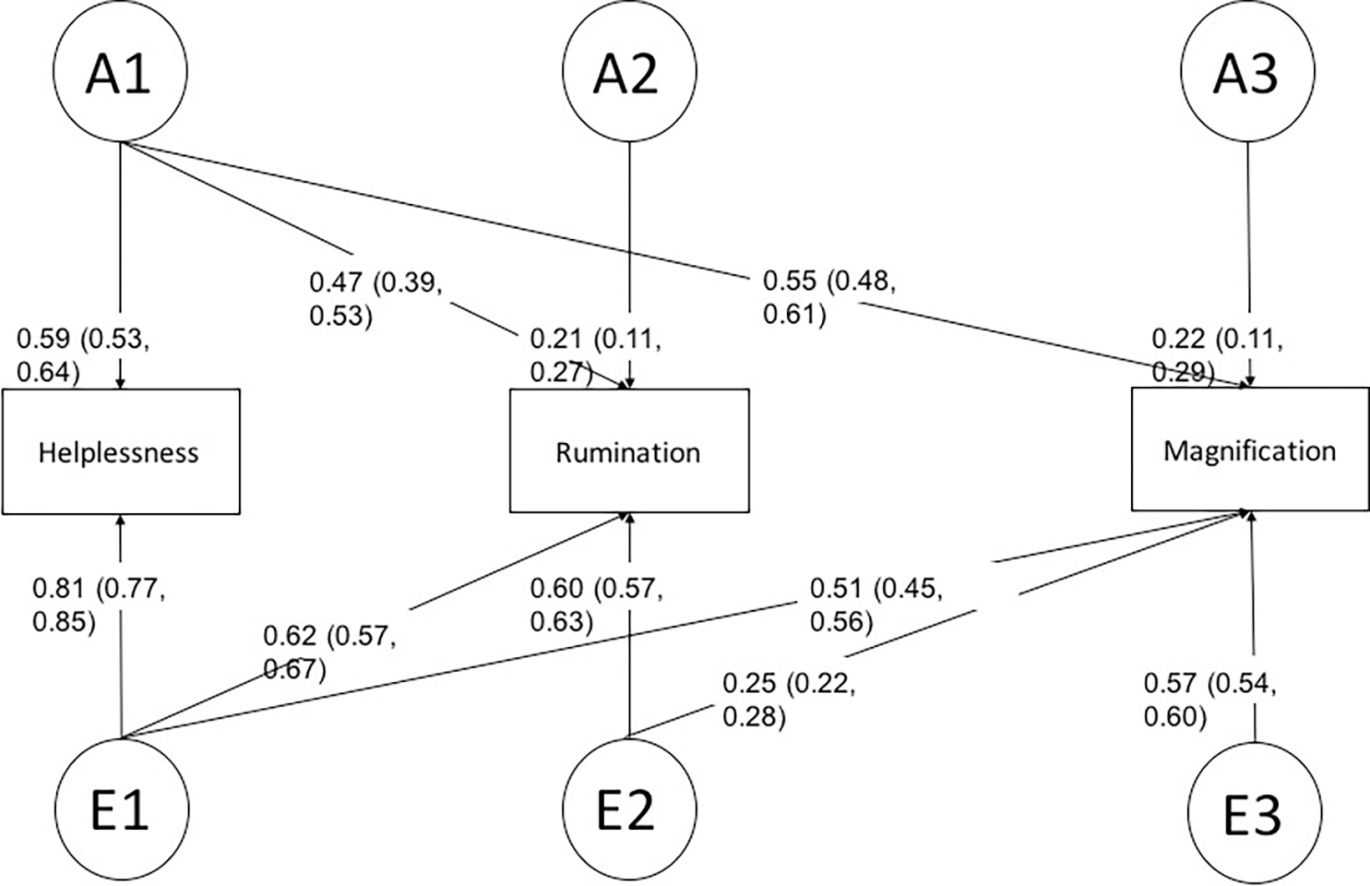
Path diagram of the best fitting AE Cholesky model depicting the sources of covariance between helplessness, rumination, and magnification of the Pain Catastrophizing Scale. The variance and covariance of the monozygotic and dizygotic female twin pairs was decomposed into additive genetic (A1-A3) and unshared environmental (E1-E3) factors. Standardized factor loadings with 95% confidence interval are displayed. Squaring the loadings and multiplying them by 100 results in the phenotypic variance explained by the specific factor.

Model comparison likelihood ratio tests showed no significant differences between the fully saturated model and the full ACE (p = 0.27) and ADE Cholesky models (p = 0.27), indicating that the assumption for twin modelling was satisfied (Table 2). Among the three full models (ACE, ADE, and the saturated model), the full ADE Cholesky model provided the best balance of model fit and parsimony, based on the AIC. Sub-model comparison based on the AIC revealed a Cholesky model without any D factors and without a path from A2 to magnification to provide the best fit to the data (Table 3; Fig 1). In this best mode, we identified a genetic factor explaining all sub-scales and genetic factors unique to rumination (A2) and magnification (A3) (Fig 1). In this model, genetic factors explained 36% of the phenotypic variance in helplessness, 28% in rumination, and 35% in magnification. Genetic and environmental correlations (i.e., overlaps in genetic and environmental effects) across the three PCS subdomains are shown in Table 4. The genetic correlations among the three PCS subdomains were very high (r_G_ = .84 to .93), indicating a large overlap in genetic effects in these phenotypes. Although also substantial, the environmental correlations among the three subdomains were consistently lower compared to the genetic correlations (r_E_ = .63 to .73; Table 4).

**Table 3:** Results of the model comparison for the three subdomains of pain catastrophizing (helplessness, magnification, rumination).

Model names	Differences of log likelihood	Differences of df	P vales of likelihood ratio tests	AIC
The fully saturated model	Reference	Reference	Reference	1481.51
The full ACE Cholesky model compared with the full saturated model	40.64	36	0.27	1450.16
The full ADE Cholesky model compared with the full saturated model	40.47	36	0.27	1449.99
**The best model (The full ADE Cholesky model without all D factors and a path from A2 to magnification. Please see Fig 1) compared with the full ADE Choesky model.**	**2.00**	**7**	**0.95**	**1437.99**

Abbreviations: A = additive genetic factors; C = shared environmental factors; D = non-additive genetic factors; E = non-shared environmental factors; AIC = Akaike information criterion; df = degree of freedom.

**Table 4:** Genetic and environmental correlations across the three PCS subdomains, as well as for pain catastrophizing, neuroticism, anxiety sensitivity, and fear of pain.

	Helplessness	Rumination	Magnification		Neuroticism	AS	Fear of Pain	PCS Total
**Genetic correlation**								
	Helplessness	Rumination	Magnification		Neuroticism	AS	Fear of Pain	PCS Total
Helplessness	1.00			Neuroticism	1.00			
Rumination	0.90 (0.89, 0.94)	1.00		AS	0.72 (0.71, 0.75)	1.00		
Magnification	0.93 (0.91, 0.98)	0.84 (0.80, 0.93)	1.00	Fear of Pain	0.61 (0.60, 0.76)	0.89 (0.88, 0.99)	1.00	
-	-	-	-	PCS Total	0.46 (0.21, 0.57)	0.93 (0.579, 0.96)	0.72 (0.524, 0.95)	1.00
**Environmental correlation**								
Helplessness	1.00			Neuroticism	1.00			
Rumination	0.73 (0.71, 0.74)	1.00		AS	0.09 (-0.02, 0.19)	1.00		
Magnification	0.63 (0.62, 0.65)	0.68 (0.66, 0.70)	1.00	Fear of Pain	0.25 (0.14, 0.32)	0.43 (0.34, 0.51)	1.00	
-	-	-	-	PCS Total	0.11 (-0.02, 0.20)	0.12 (0.01, 0.23)	0.20 (0.01, 0.34)	1.00

AS = Anxiety Sensitivity; PCS = Pain Catastrophizing Scale. Genetic and Environmental correlations show overlaps in genes and environmental effects and range from– 1 to +1. When the genetic correlation is +1, the two sets of genes overlap completely.

### Multivariate modelling of pain catastrophizing and related measures of negative affect and personality

**Fig 2:**
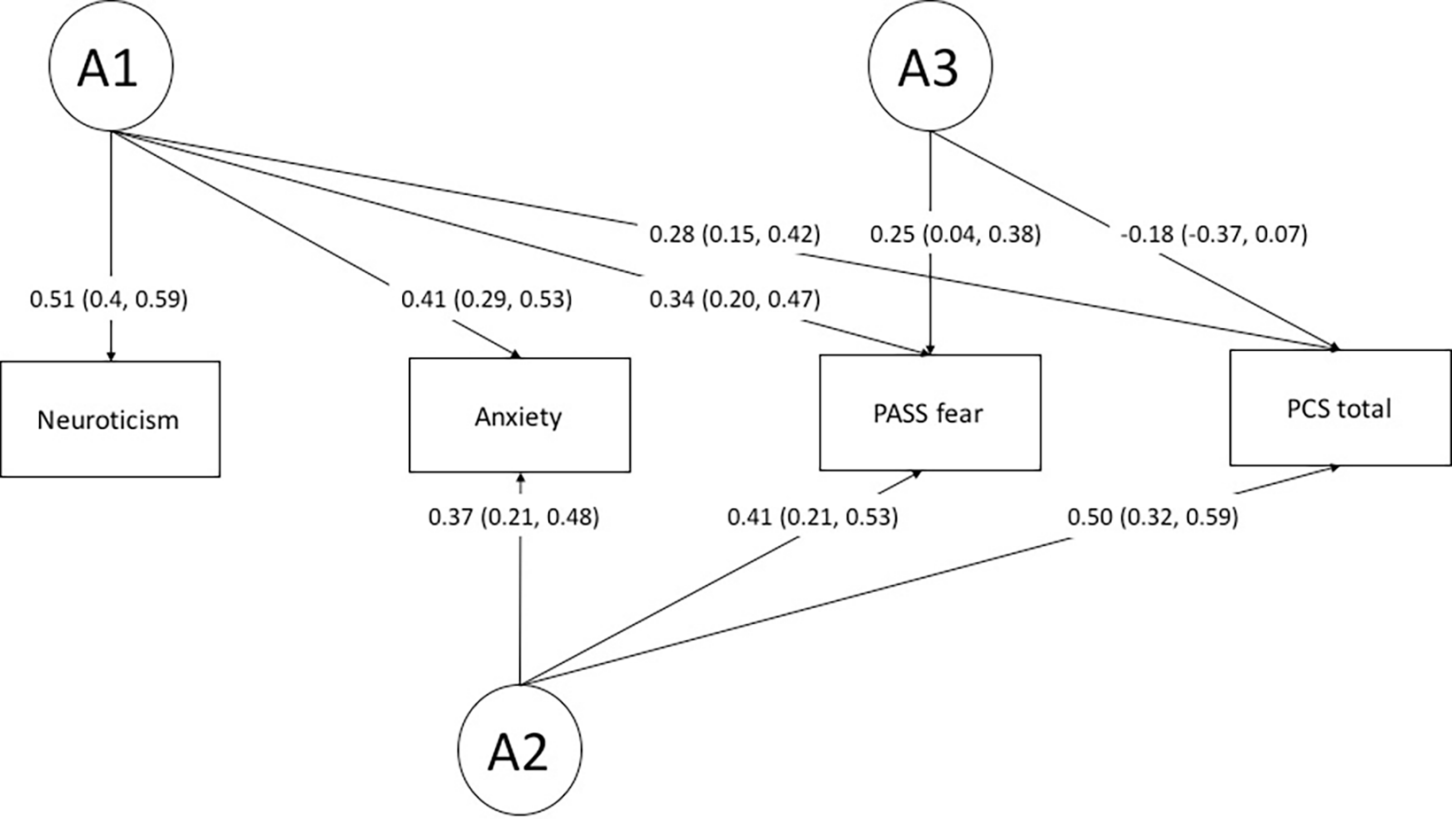
Path diagram of the best fitting AE Cholesky model depicting the sources of additive genetic variance and covariance (A1-A3) between neuroticism, anxiety sensitivity, fear of pain, and pain catastrophizing. Standardized factor loadings with 95% confidence interval are displayed. Squaring the loadings and multiplying them by 100 results in the phenotypic variance explained by the specific additive genetic factor.

**Fig 3:**
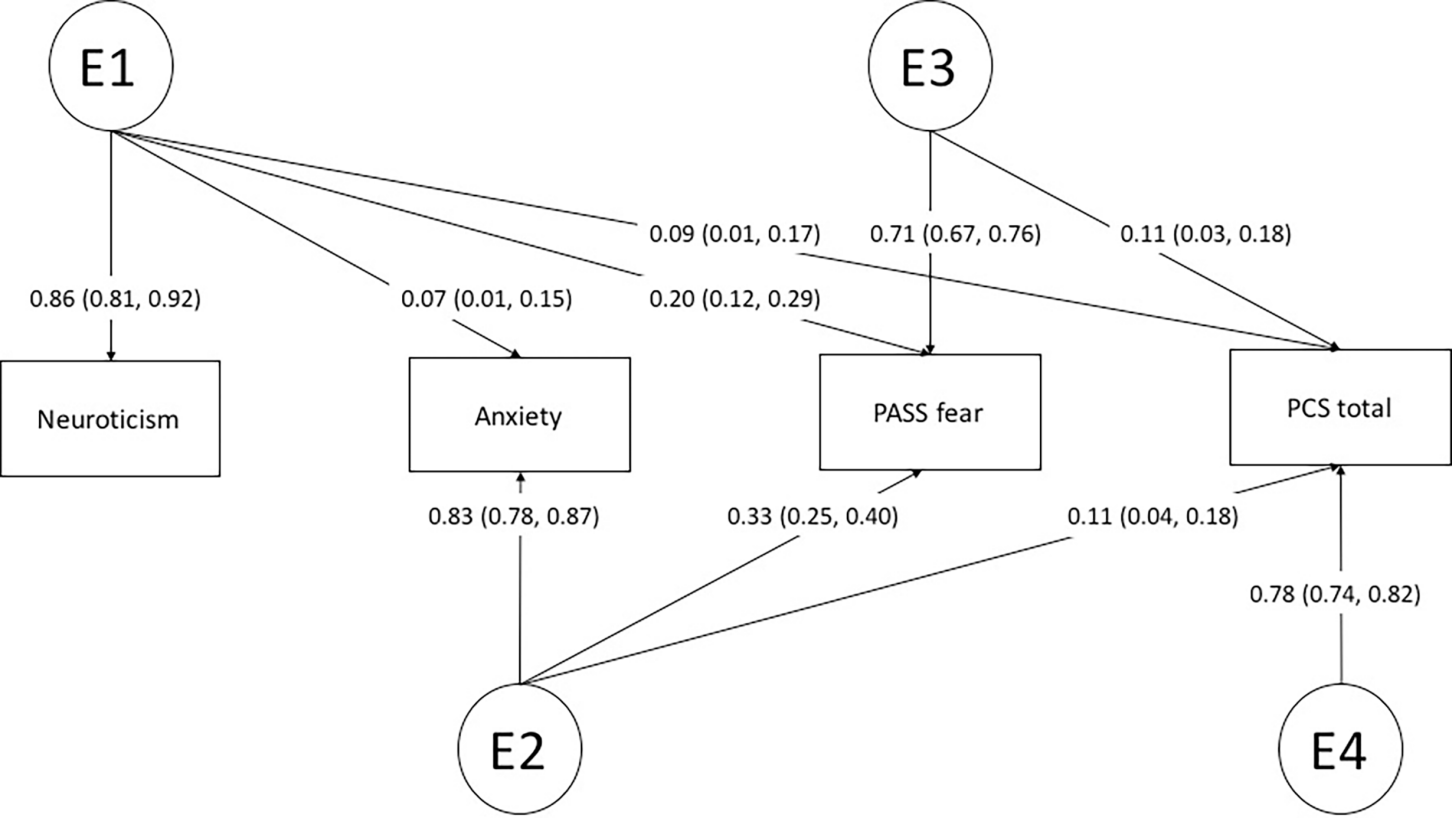
Path diagram of the best fitting AE Cholesky model depicting the sources of environmental variance and covariance (E1-E3) between neuroticism, anxiety sensitivity, fear of pain, and pain catastrophizing. Standardized factor loadings with 95% confidence interval are displayed. Squaring the loadings and multiplying them by 100 results in the phenotypic variance explained by the specific environmental factor.

The fully saturated model did not differ significantly from the full ACE (p = 0.15) and the ADE Cholesky model (p = 0.19), again indicating that the assumption for twin modelling was satisfied (Table 5). Among the three full models (ACE, ADE, and the fully saturated), the full ADE Cholesky model provided the best fit to the data based on the AIC. ADE submodel comparison revealed a Cholesky model without any influence of D and without a path from the genetic factor A4 to pain catastrophizing to be the most parsimonious model (Table 5; Figs 2 and 3).

**Table 5:** Results of the model comparison for pain catastrophizing, neuroticism, anxiety sensitivity, and fear of pain among the full ADE Cholesky model and the sub-models.

Model names	Differences of log likelihood	Differences of df	P vales of likelihood ratio tests	AIC
The fully saturated model	Reference	Reference	Reference	4565.75
The full ACE Cholesky model compared with the fully saturated model	66.92	58	0.15	4518.80
The full ADE Cholesky model compared with the fully saturated model	69.05	58	0.19	4516.67
The best model (the full ADE Cholesky model without all D factors and a path from A4 to PCS) compared with the full ADE Cholesky model	4.39	11	0.95	4499.06

Abbreviations: A = additive genetic factors; D = non-additive genetic factors; E = non-shared environmental factors; AIC = Akaike information criterion; df = degree of freedom; PCS = Pain Catastrophizing Scale.

In the best model, three genetic factors could be detected, with one genetic factor (A1) loading on all phenotypes, one (A2) loading on anxiety sensitivity, fear of pain, and pain catastrophizing, and one (A3) loading on fear of pain and pain catastrophizing only (Fig 2). In contrast, four environmental factors could be detected (E1-E4) with one (E1) loading on all phenotypes although highest on neuroticism. E2 loaded highest on anxiety sensitivity (0.82) and E3 highest on fear of pain (0.72). Furthermore, an environmental factor loading on pain catastrophizing only could be detected, explaining 61% of the phenotypic variance (calculated by squaring the factor loads and multiplying the result by 100). The genetic correlations across neuroticism, anxiety sensitivity, fear of pain, and pain catastrophizing indicated a large genetic overlap between anxiety sensitivity and pain catastrophizing (r_G_ = 0.93) a more modest overlap between fear of pain and pain catastrophizing (r_G_ = 0.72) and a modest overlap between pain catastrophizing and neuroticism (r_G_ = 0.46) (Table 4). The environmental correlations were substantially lower, ranging from r_E_ = .11 to .20, indicating considerably fewer shared environmental influences.

## Discussion

In the present study we report moderate heritabilities of pain catastrophizing (36%) and its three subdomains of helplessness (35%), rumination (27%), and magnification (36%) in women. This work confirms an earlier study from the US that reported remarkably similar heritability estimates of pain catastrophizing [21] as well as providing the first evidence that the subdomains of helplessness, magnification and rumination are all significantly heritable, largely to the same extent. The high correlations observed between the three subdomains of pain catastrophizing were mainly explained by overlapping genetic factors, with one common factor loading on all three subdomains. Although the three subdomains also showed some overlapping environmental sources, no effects of common environment (e.g. upbringing) on pain catastrophizing or any of its subdomains could be detected. In addition we also report, for the first time, high genetic correlations between pain catastrophizing and other measures of negative affect such as fear of pain and anxiety sensitivity. In contrast, a much lower genetic overlap was found between neuroticism and pain catastrophizing. Finally our study results also show that shared environmental factors likely play a minor role in the relationship between pain catastrophizing and other measures of negative affect and personality.

### Pain catastrophizing and subdomains helplessness, rumination, and magnification

Continued conceptual and measurement advances have refined pain catastrophizing as a multifaceted construct, where in addition to the original domain of helplessness, two additional domains of magnification and rumination have been added. Rather than focusing solely on the predictive role of the overall construct, recent research has started to investigate the differential impact of the specific aspects of pain catastrophizing on pain outcomes. For example, in a study by Craner et al., the authors found helplessness to be an independent predictor of pain severity, pain-related interference, as well as mental and physical health-related quality of life, and depressed mood, whereas magnification was significantly related to physical and mental health-related quality of life and depressed mood only, and rumination showed no unique association with any of these outcomes [30]. Another study examined the unique effects of the three subdomains on changes in pain outcomes in chronic pain patients undergoing an interdisciplinary pain rehabilitation program and found helplessness and rumination to be particularly useful targets in improving treatment outcomes [31]. Finally, a recent study has shown that rumination, but not magnification or helplessness, may help to mediate sex and ethnicity related differences in pain tolerance [32].

In accordance with these findings we also report evidence for phenomenological differences between the three subdomains of pain catastrophizing, i.e. specific genetic factors for rumination and magnification could be identified. This supports previous findings that different single nucleotide polymorphisms related to serotonergic transmission may be associated with different subdomains of pain catastrophizing [27]. However, a shared genetic factor loading on all three subdomains could also be observed in our study, perhaps helping to explain the high correlations often observed between these variables. Interestingly, while unique environmental factors explained more of the overall phenotypic variance compared to genetic factors, the environmental correlations between the subdomains of pain catastrophizing were consistently lower compared to the genetic correlations. In other words, genetic factors contribute more to the shared variance across the three subdomains of pain catastrophizing compared to environmental effects. Overall, our results support the retention of the current three factor model of pain catastrophizing, indicating some important aetiological differences between the three subdomains of magnification, rumination and helplessness but also suggesting that there is a shared genetic predisposition to all of them. Of interest, we could find no common environmental effects such as upbringing on pain catastrophizing or any of its subdomains [18–20].

### Pain catastrophizing and its link with personality and negative affect

We demonstrated a very strong genetic correlation between pain catastrophizing and anxiety sensitivity (0.93) and, to a slightly lesser degree, between pain catastrophizing and fear of pain (0.72). On the basis of our results, clinicians should be aware that individuals showing high levels of anxiety sensitivity or fear of pain have a stronger genetic predisposition to develop pain catastrophizing tendencies (and perhaps vice versa). The genetic correlation between pain catastrophizing and neuroticism was considerable weaker in comparison to the other measures, albeit still significant. Overall, the genetic correlations between pain catastrophizing, anxiety sensitivity, fear of pain, and neuroticism were much higher in comparison to the low environmental correlations between the phenotypes. Of particular interest we found no evidence of common environmental factors such as upbringing in the relationship between these measures. From an aetiological point of view, this indicates that while the psychological constructs share similar genetic influences, the environmental factors that may lead to their expression are not necessarily the same (Table 4). In fact, 61% of the variance in pain catastrophizing could be explained by unique environmental factors that were not shared with the other phenotypes. These findings support the measurement of pain catastrophizing as an important psychological construct distinct from other measures of negative affect and personality. Furthermore, they suggest that different preventative and treatment strategies may be needed to adequately target each of these constructs.

### Limitations

The results should be interpreted in view of some limitations. First, we used a population sample of individuals which might show lower variability of pain catastrophizing than individuals recruited from a clinic. Genetic studies in clinical samples might yield different heritabilities and results. Thus, these findings should not be extrapolated to other samples, especially not clinical ones. Second, because of the relatively small fraction of men for which data were available, the here discussed findings do only apply to women and cannot be extrapolated to men. Results from the multivariate twin analyses conducted on a small sample of men (N = 332) are presented as supporting information (S1–S5 Tables, S1 and S2 Figs) but because of low statistical power these were not included in the main analyses and are therefore not further elaborated on. More in depth research in males is warranted. Sex differences in pain catastrophizing, as well as anxiety sensitivity and fear of pain have been previously reported and should ideally be addressed in future studies [1,33]. Finally, pain catastrophizing, negative affect, as well as their associations may vary with age and although we controlled for the main effects of age in the models using SEM, extrapolation of the results to other age groups should not be made. Ideally, longitudinal study designs will be needed to get a better understanding of the age-dependent phenotypic variation.

In conclusion, this is the first study to suggest shared genetic factors in the observed association between pain catastrophizing and negative affect (i.e., anxiety sensitivity and fear of pain). In addition, we provide further evidence for a genetic basis for pain catastrophizing and, for the first time, its three subdomains rumination, helplessness, and magnification. Our findings provide new insights into the aetiology of pain catastrophizing and its relationship with other measures of negative affect and personality. Pain catastrophizing remains one of the most important psychological predictors of pain intensity, disability and treatment outcomes across a range of musculoskeletal and rheumatological conditions. Every effort should be made to better understand the factors contributing to its development and how best to address it clinically.

## Materials and methods

### Participants

Participants were drawn from the TwinsUK registry, a cohort of over 13,000 male and female adult twins from the UK. A detailed description of the registry, the participant recruitment procedures, and the cohort’s comparability with singleton populations can be found elsewhere [34–36]. Information on pain catastrophizing was collected electronically between June and August 2016. A subsample of twins for whom a contact email address was available and who had previously stated their willingness to be included in future pain-related research were invited to participate in this study. Furthermore, only twins >18 years and for which zygosity had been previously established were considered. Of a total of 5906 twins who were contacted, 3061 completed the full questionnaire (response rate of 51.8%). Information on anxiety sensitivity, personality, and fear of pain was available from previous surveys.

To minimise ethnic heterogeneity, self-reported non-Caucasian individuals (N = 291) were excluded from the analyses. In addition, opposite-sex twin pairs (N = 14 pairs), twins with unknown zygosity (N = 9), and 332 men were also excluded. The final sample of N = 2401 female participants consisted of 62% (N = 1491) monozygotic (MZ) twins and 38% (N = 910) dizygotic (DZ) twins, including 497 full MZ pairs, 246 full DZ pairs, and 915 individuals whose co-twin did not participate. All twins in the sample had been reared together. Twin pairs where one twin had data but the co-twin did not have data (treated as missing values) were also included in the study. For all twins, zygosity had been previously assigned using a standard questionnaire and had been confirmed with multiplex DNA genotyping and, more recently, by means of genetic association markers on DNA obtained from venous blood samples. The present study received approval from the ethics committee at St. Thomas´ Hospital, and written consent was obtained before data collection. The research followed the tenets of the Declaration of Helsinki.

### Material

#### Pain catastrophizing

Information on pain catastrophizing was collected using the “Pain Catastrophizing Scale” (PCS) [22]. In the PCS, participants are asked to reflect on past painful experiences and to indicate the degree to which they experienced certain thoughts or feelings when experiencing pain. Response options for the 13 items are on a 5-point Likert-type scale ranging from (0) not at all to (4) all the time. A total score, as well as three subscales scores (for rumination, magnification, and helplessness) may be computed by summing up the item scores. The PCS has been shown to have good psychometric properties and adequate internal consistency [22,37–38]. Cronbach’s α in our study was .94 for total PCS, .89 for helplessness, .74 for magnification, and .92 for rumination.

#### Anxiety sensitivity

Anxiety sensitivity is defined as the fear of arousal-related sensations (e.g., fear of heart palpitations), arising from beliefs that these anxiety-related sensations have harmful consequences [39]. Information on anxiety sensitivity was assessed using the self-report “Anxiety Sensitivity Index” (ASI) [39]. All 16 questionnaire items are responded to on a 5-point Likert-type scale ranging from very little (0) to very much (4). The sum of all ASI responses yields the total ASI score. The instrument has repeatedly shown excellent psychometric properties and predictive validity, as well as internal consistency (a = 0.81–0.94), a good degree of test/retest reliability (r = 0.71–0.75), and a high degree of inter-item relatedness [40]. Cronbach’s α in our study was 0.89.

#### Neuroticism

Neuroticism was assessed using the two corresponding items of the “Ten-Item Personality Index” (TIPI) [41]. The TIPI is a short 10-item questionnaire assessing the big five dimensions of extroversion, agreeableness, conscientiousness, emotional stability, and openness to experiences. Response options are on a 7-point Likert scale ranging from disagree strongly (1) to agree strongly (7). Dimension scores are created by summing the two item values for the different dimensions. The questionnaire has been designed to measure very broad domains with only two items per dimension and by using items at both the positive and negative poles. Hence, the use of the TIPI is indicated mainly for situations where short measures are needed and personality is not the primary topic of interest. Despite its brevity, the instrument has shown adequate convergence with widely used multi-item big-five measures (e.g., BFI) in self-, observer and peer reports (mean of r = 0.77), as well as good test-retest reliability (r = 0.62–0.77) [42]. Cronbach’s α for neuroticism in our study was 0.54.

#### Fear of pain

For the assessment of fear of pain (i.e., fearful thoughts about pain or its consequences), the corresponding subscale of the “Pain Anxiety Symptoms Scale -20” (PASS-20) was used [43]. The PASS-20 is a self-report instrument measuring pain-specific anxiety symptoms and consists of four 5-item subscales measuring cognitive anxiety responses, escape and avoidance, fearful thinking and physiological anxiety responses. All items are rated on a frequency scale from 0 (never) to 5 (always). Subdomain scores are computed by summing the ratings across the subscale items. The psychometric properties of the PASS-20 have been evaluated on 282 chronic pain patients, where it showed strong internal consistency (α = 0.75 to 0.91), high reliability (r = 0.81 to 0.89) and good predictive and construct validity [44]. Cronbach’s α in our study of the fearful thinking subscale in the present study was 0.79.

### Statistical methods

Non-genetic data handling and analyses were undertaken using STATA (Version 14; StataCorp. 2015. *Stata Statistical Software*: *Release 14*. College Station, TX: StataCorp LP). Genetic analyses were conducted using the R package “OpenMx”. For the multivariate genetic analyses, R statistical software, version 3.1.2 was used (R Core Team, 2014). Normal distribution of the variables was assessed by visual inspection of the histograms and by performing Shapiro–Wilk tests. Descriptive statistics for the overall sample and by zygosity were calculated as means and standard deviations for continuous measures and as percentages for categorical measures. To account for non-normality of the data, Mann-U Whitney tests (for continuous measures) and chi^2^ tests (for categorical and dichotomous measures) were used to compare MZ and DZ twins for systematic differences across the study variables. To investigate the phenotypic associations between our variables of interest, Spearman correlations were computed. All tests were two-tailed. For all analyses, a P value less than 0.05 was considered statistically significant, unless stated otherwise.

### Twin design and modelling

The classical twin design rests on two main assumptions: 1. MZ twins derive from a single fertilized egg and share identical genotypes (i.e.,> 99.9%), whereas DZ twins are no more genetically alike than siblings, sharing on average 50% of their segregating genes. 2. MZ and DZ twin pairs share the intra-pair environment to the same degree [45]. These specific design properties allow for the adjustment of all measured and unmeasured genetic and environmental similarities that make MZ twins similar to one another. Based on the second assumption, a higher MZ than DZ correlation in the phenotype of interest provides a first impression of the magnitude of the genetic influence [46]. This is also called heritability (h^2^). To estimate the heritability of pain catastrophizing in the present study, we used structural equation modelling (SEM) to apportion the total phenotypic variance into additive genetic (A), non-linear genetic (or dominant genetic—D) common environmental (C), and unique environmental (E) components [47]. A represents the additive effects of alleles at the relevant genetic loci and is assumed to be perfectly correlated in MZ pairs while being correlated at 0.5 in DZ pairs; C represents environmental influences that make twins raised together more similar and is assumed to be perfectly correlated for both MZ and DZ pairs; E represents experiences that are unique to each twin in a pair, are completely uncorrelated for both MZ and DZ pairs, and that therefore drives within-pair differences. E further includes measurement error. In cases where the MZ correlation is more than twice the DZ correlation, an alternative model can be fitted where the C component is dropped and instead non-linear genetic effects are included.

In the present study, univariate genetic modelling was extended to multivariate model fitting to explore to what degree the same genetic and environmental factors contribute to the phenotypic covariation (i.e., inter-correlations) between the phenotypes included in the model (i.e. pain catastrophizing, anxiety sensitivity, fear of pain, and neuroticism). For this, cross-trait cross-twin correlations in MZ and DZ were computed [47,48]. CTCTs that are greater in MZ than DZ twins are indicative of genetic factors contributing to the phenotypic correlations between the two variables.

To identify genetic and environmental structure behind phenotypic associations among the variables, multivariate twin modelling was performed using SEM with a fully saturated model and a number of Cholesky models. The fully saturated model treats variance-covariance matrix as a free parameter equivalent to the sample variance-covariance matrix. The Cholesky model decomposes the variance and covariance of the observed study variables (i.e., PCS total score, neuroticism, anxiety sensitivity, and fear of pain) into latent variables (i.e., A, C or D, and E factors) by equating the mean and variance of each observed variable across twin order and zygosity.

First, we compared the full ACE and ADE Cholesky model to the fully saturated model. Using a likelihood ratio test, this model comparison tests whether the assumption for twin modelling is satisfied and indicates which factor (C or D) is more appropriate to be included in the model according to the Akaike information criterion (AIC), given that C and D cannot be included in the same model when using SEM. Models with lower AICs are considered more parsimonious and therefore a better representation of the data. In a second step, the full Cholesky models (ACE and ADE) were compared to the full AE Cholesky model to test whether C or D is significant by means of likelihood ratio test and according to AIC [48,49] Based on the most plausible model (either ACE, ADE, or A), sub-models were created by subsequently dropping path coefficients (i.e., A, C or D, and E), or by eliminating some of the latent variables. Those sub-models were compared with each other based on their AIC, and with the full ACE or ADE Cholesky models by means of likelihood ratio test and AIC.

For model comparison and to obtain standardized path coefficients with 95% confidence intervals (CIs), SEM with full information maximum-likelihood estimation was performed. FIML method was used to deal with missing values with less bias [50]. Even when there are several missing values in a row (e.g., a twin pair), FIML can calculate likelihood by using the other information of the row (e.g., the twin pair) except for the missing values. Thus, the sample size of all twin models was the same (n = 2401). FIML is often used in both structural equation modelling and quantitative-genetic models [50]. A twin pair of which a twin participated in the study, but the other twin did not participate, can be also included into the genetic twin models. Using logistic regression, we first tested whether the missing values could be predicted by sociodemographic or any of the study variables. None of them turned out to be significant, hence validating the assumption that missing values followed a missing completely at random mechanism. Under the missing-at-random assumption, full-information maximum likelihood was implemented to handle missing values and give preferred parameter estimates. All observed variables were adjusted for age in all of the models.

## Supporting information

S1 Fig Path diagram of the best fitting AE Cholesky model depicting the sources of additive genetic variance and covariance (A1-A3) between neuroticism, anxiety sensitivity, fear of pain, and pain catastrophizing in a subsample of men (N = 332). Standardized factor loadings with 95% confidence interval are displayed. Squaring the loadings and multiplying them by 100 results in the phenotypic variance explained by the specific additive genetic factor.
(TIFF)

S2 Fig Path diagram of the best fitting AE Cholesky model depicting the sources of environmental variance and covariance (E1-E3) between neuroticism, anxiety sensitivity, fear of pain, and pain catastrophizing in a subsample of men (N = 332). Standardized factor loadings with 95% confidence interval are displayed. Squaring the loadings and multiplying them by 100 results in the phenotypic variance explained by the specific environmental factor.
(TIFF)

S1 Table Sociodemographic characteristics and main study variables of the overall sample of men (N = 332) and by zygosity. (DOCX)

S2 Table Phenotypic correlations between the main study variables in the male subsample (N = 332). (DOCX)

S3 Table Results of the model comparison for the three subdomains of pain catastrophizing (helplessness, magnification, rumination) in a subsample of men (N = 332). (DOCX)

S4 Table Genetic and environmental correlations across the three PCS subdomains, as well as for pain catastrophizing, neuroticism, anxiety sensitivity, and fear of pain in a subsample of men (N = 332). (DOCX)

S5 Table Results of the model comparison for pain catastrophizing, neuroticism, anxiety sensitivity, and fear of pain among the full Cholesky models and the sub-models in a subsample of men (N = 332). (DOCX)
