# Supplementary material for: Pain catastrophizing, neuroticism, fear of pain, and anxiety: Defining the genetic and environmental factors in a sample of female twins
Source: PLoS One. 2018 Mar 22;13(3):e0194562. doi: 10.1371/journal.pone.0194562 (PMC5864012; doi:10.1371/journal.pone.0194562)
Supplement: S1 Table — (DOCX) [file pone.0194562.s003.docx]

**Supporting Table 1** Sociodemographic characteristics and main study variables of the overall sample of men (N = 332) and by zygosity.

|  | Full sample  (N =332) | | MZ  (N = 237) | | DZ  (N = 95) | |  |  |
| --- | --- | --- | --- | --- | --- | --- | --- | --- |
|  | **N** | **%** | **N** | **%** | **N** | **%** |  |  |
|  | **M** | **SD** | **M** | **SD** | **M** | **SD** | **z** | ***P*** |
| Age | 58.43 | 15.40 | 56.20 | 16.11 | 63.98 | 11.84 | -4.355 | 0.000** |
| PCS total | 8.37 | 7.99 | 7.92 | 7.54 | 9.50 | 8.98 | -1.190 | 0.234 |
| Rumination | 3.43 | 3.42 | 3.25 | 3.23 | 3.87 | 3.84 | -0.914 | 0.361 |
| Magnification | 1.81 | 1.91 | 1.79 | 1.89 | 1.87 | 1.97 | -0.277 | 0.782* |
| Helplessness | 3.12 | 3.59 | 2.87 | 3.36 | 3.75 | 4.05 | -1.789 | 0.074 |
| Anxiety Sensitivity | 11.83 | 8.09 | 11.72 | 7.94 | 12.05 | 8.43 | -0.067 | 0.947 |
| Neuroticism | 2.85 | 1.23 | 2.91 | 1.24 | 2.72 | 1.21 | 0.829 | 0.497 |
| Fear of pain | 3.73 | 3.76 | 3.62 | 3.61 | 3.94 | 4.07 | -0.150 | 0.881 |

* p-value < .05; ** p-value < 0.001

M = Mean; SD = Standard Deviation; PCS = Pain Catastrophizing Scale.
