# Supplementary material for: Pain catastrophizing, neuroticism, fear of pain, and anxiety: Defining the genetic and environmental factors in a sample of female twins
Source: PLoS One. 2018 Mar 22;13(3):e0194562. doi: 10.1371/journal.pone.0194562 (PMC5864012; doi:10.1371/journal.pone.0194562)
Supplement: S2 Table — (DOCX) [file pone.0194562.s004.docx]

**Supporting Table 2** Phenotypic correlations between the main study variables in the male subsample (N = 332).

|  | PCS total | Rumination | Magnification | Helplessness | Anxiety Sensitivity | Neuroticism | Fear of pain |
| --- | --- | --- | --- | --- | --- | --- | --- |
| PCS total | - | - | - | - | - | - | - |
| Rumination | 0.91* | - | - | - | - | - | - |
| Magnification | 0.81* | 0.63* | - | - | - | - | - |
| Helplessness | 0.92* | 0.73* | 0.67* | - | - | - | - |
| Anxiety Sensitivity | 0.46* | 0.38* | 0.51* | 0.38* | - | - | - |
| Neuroticism | 0.25* | 0.18* | 0.27* | 0.25* | 0.34* | - | - |
| Fear of pain | 0.38* | 0.28* | 0.45* | 0.34* | 0.48* | 0.27* | - |

* p-value < .001; PCS = Pain Catastrophizing Scale.
