# Supplementary material for: Pain catastrophizing, neuroticism, fear of pain, and anxiety: Defining the genetic and environmental factors in a sample of female twins
Source: PLoS One. 2018 Mar 22;13(3):e0194562. doi: 10.1371/journal.pone.0194562 (PMC5864012; doi:10.1371/journal.pone.0194562)
Supplement: S3 Table — (DOCX) [file pone.0194562.s005.docx]

**Supporting Table 3** Results of the model comparison for the three subdomains of pain catastrophizing (helplessness, magnification, rumination) in a subsample of men (N = 332).

| Model names | Differences of log likelihood | Differences of df | P vales of likelihood ratio tests | AIC |
| --- | --- | --- | --- | --- |
| The fully saturated model | Reference | Reference | Reference | 391.35 |
| The full ACE Cholesky model compared with the full saturated model | 40.15 | 36 | 0.291 | 359.50 |
| The full ADE Cholesky model compared with the full saturated model | 40.90 | 36 | 0.263 | 360.25 |
| **The best model (The full ACE Cholesky model without all C factors and a path from A2 to magnification. Please see Figure 1) compared with the full ACE Choesky model.** | **4.23** | **9** | **0.895** | **345.74** |

Abbreviations: A = additive genetic factors; C = shared environmental factors; D = non-additive genetic factors; E = non-shared environmental factors; AIC = Akaike information criterion; df = degree of freedom
