# Supplementary material for: Pain catastrophizing, neuroticism, fear of pain, and anxiety: Defining the genetic and environmental factors in a sample of female twins
Source: PLoS One. 2018 Mar 22;13(3):e0194562. doi: 10.1371/journal.pone.0194562 (PMC5864012; doi:10.1371/journal.pone.0194562)
Supplement: S4 Table — (DOCX) [file pone.0194562.s006.docx]

**Supporting Table 4** Genetic and environmental correlations across the three PCS subdomains, as well as for pain catastrophizing, neuroticism, anxiety sensitivity, and fear of pain in a subsample of men (N = 332).

|  | Helplessness | Rumination | Magnification |  | Neuroticism | AS | Fear of Pain | PCS Total |
| --- | --- | --- | --- | --- | --- | --- | --- | --- |
| **Genetic correlation** | | | | | | | | |
|  | Helplessness | Rumination | Magnification |  | Neuroticism | AS | Fear of Pain | PCS Total |
| Helplessness | 1.00 |  |  | Neuroticism | 1.00 |  |  |  |
| Rumination | 1.00 | 1.00 |  | AS | 1.00 | 1.00 |  |  |
| Magnification | 1.00 | 1.00 | 1.00 | Fear of Pain | 0.81 (-0.65, 0.79) | 0.81 (0.65, 0.79) | 1.00 |  |
| - | - | - | - | PCS Total | 0.59  (-0.13, 0.71) | 0.59 (0.13 0.71) | 0.95 (-0.68, 0.99) | 1.00 |
| **Environmental correlation** | | | | | | | | |
| Helplessness | 1.00 |  |  | Neuroticism | 1.00 |  |  |  |
| Rumination | 0.66 (0.60, 0.70) | 1.00 |  | AS | 0.19 (-0.07, 0.37) | 1.00 |  |  |
| Magnification | 0.55  (0.48, 0.58) | 0.55 (0.39, 0.63) | 1.00 | Fear of Pain | 0.27 (0.00, 0.41) | 0.35 (0.07, 0.56) | 1.00 |  |
| - | - | - | - | PCS Total | 0.25 (0.02, 0.36) | 0.30 (0.08, 0.50) | 0.16 (0.01, 0.32) | 1.00 |

AS = Anxiety Sensitivity; PCS = Pain Catastrophizing Scale. Genetic and Environmental correlations show overlaps in genes and environmental effects and range from – 1 to +1. When the genetic correlation is +1, the two sets of genes overlap completely.
