# Supplementary material for: Pain catastrophizing, neuroticism, fear of pain, and anxiety: Defining the genetic and environmental factors in a sample of female twins
Source: PLoS One. 2018 Mar 22;13(3):e0194562. doi: 10.1371/journal.pone.0194562 (PMC5864012; doi:10.1371/journal.pone.0194562)
Supplement: S5 Table — (DOCX) [file pone.0194562.s007.docx]

**Supporting Table 5** Results of the model comparison for pain catastrophizing, neuroticism, anxiety sensitivity, and fear of pain among the full Cholesky models and the sub-models in a subsample of men (N = 332).

| Model names | Differences of log likelihood | Differences of df | P vales of likelihood ratio tests | AIC |
| --- | --- | --- | --- | --- |
| The fully saturated model | Reference | Reference | Reference | 644.03 |
| The full ACE Cholesky model compared with the fully saturated model | 64.5 | 58 | 0.260 | 592.52 |
| The full ADE Cholesky model compared with the fully saturated model | 64.7 | 58 | 0.254 | 592.73 |
| The best model (the full ACE Cholesky model without all C factors, A2 and A4 factors, a path from E3 to PCS) compared with the full ACE Cholesky model | 2.04 | 15 | 0.999 | 564.56 |

Abbreviations: A = additive genetic factors; C=shared environmental factors; D = non-additive genetic factors; E = non-shared environmental factors; AIC = Akaike information criterion; df = degree of freedom; PCS = Pain Catastrophizing Scale.
